# Detecting cancer clusters in a regional population with local cluster tests and Bayesian smoothing methods: a simulation study

**DOI:** 10.1186/1476-072X-12-54

**Published:** 2013-12-07

**Authors:** Dorothea Lemke, Volkmar Mattauch, Oliver Heidinger, Edzer Pebesma, Hans-Werner Hense

**Affiliations:** 1 Institute of Epidemiology and Social Medicine, Medical Faculty, Westfälische Wilhelms-Universität Münster, Albert-Schweitzer-Campus 1 D3, D 48149, Münster, Germany; 2 Institute for Geoinformatics, Geosciences Faculty, Westfälische Wilhelms Universität Münster, Münster, Germany; 3 Epidemiological Cancer Registry North Rhine-Westphalia, Münster, Germany

**Keywords:** Spatial cancer cluster, Local cluster tests, R, DCluster, Bayesian smoothing methods, Simulation design, Epidemiological cancer registry

## Abstract

**Background:**

There is a rising public and political demand for prospective cancer cluster monitoring. But there is little empirical evidence on the performance of established cluster detection tests under conditions of small and heterogeneous sample sizes and varying spatial scales, such as are the case for most existing population-based cancer registries. Therefore this simulation study aims to evaluate different cluster detection methods, implemented in the open soure environment R, in their ability to identify clusters of lung cancer using real-life data from an epidemiological cancer registry in Germany.

**Methods:**

Risk surfaces were constructed with two different spatial cluster types, representing a relative risk of RR = 2.0 or of RR = 4.0, in relation to the overall background incidence of lung cancer, separately for men and women. Lung cancer cases were sampled from this risk surface as geocodes using an inhomogeneous Poisson process. The realisations of the cancer cases were analysed within small spatial (census tracts, N = 1983) and within aggregated large spatial scales (communities, N = 78). Subsequently, they were submitted to the cluster detection methods. The test accuracy for cluster location was determined in terms of detection rates (DR), false-positive (FP) rates and positive predictive values. The Bayesian smoothing models were evaluated using ROC curves.

**Results:**

With moderate risk increase (RR = 2.0), local cluster tests showed better DR (for both spatial aggregation scales > 0.90) and lower FP rates (both < 0.05) than the Bayesian smoothing methods. When the cluster RR was raised four-fold, the local cluster tests showed better DR with lower FPs only for the small spatial scale. At a large spatial scale, the Bayesian smoothing methods, especially those implementing a spatial neighbourhood, showed a substantially lower FP rate than the cluster tests. However, the risk increases at this scale were mostly diluted by data aggregation.

**Conclusion:**

High resolution spatial scales seem more appropriate as data base for cancer cluster testing and monitoring than the commonly used aggregated scales. We suggest the development of a two-stage approach that combines methods with high detection rates as a first-line screening with methods of higher predictive ability at the second stage.

## Background

The introduction of a prospective and systematic cluster monitoring has been debated in Germany for a long time [1]. The German state of Lower Saxony is currently considering the introduction of such a monitoring system because unexplained incidence elevations have been observed for various cancer sites in the municipality of Asse which hosts a nuclear waste repository [2]. It is current practice in the German epidemiological cancer registries that only “event related” cluster investigations are conducted. These respond to requests from the public, from medical doctors or health departments and arise on the basis of suspected putative cancer clusters in certain, mostly small, spatial areas. Statistical testing in these cases usually involves the estimation of standardized incidence ratios (SIR), that is, the ratio of the cases of a certain malignant entity in a given area in relation to the number expected on the basis of the rates for this cancer type in an appropriate reference population. If the SIR rise is statistically significant, a cluster is suspected and further investigation is needed to verify an association with a specific source of exposure [3].

A cluster is commonly defined as a geographically confined group of cancer cases of sufficient size that are unlikely to have occurred by chance [4]. However, this approach has serious methodological limitations: On the one hand, no hypothesis driven analyses are possible since the clusters are detected before the hypothesis of elevated cancer risk areas is formulated (also known as Texas sharpshooter fallacy) [5]. On the other hand, there is a substantial multiple testing problems given the multitude of tests (different communities, different time periods, different cancer diagnoses) that must be performed. More importantly, such event-driven cluster investigations rarely discover smaller or weaker exposure related clusters nor do they help to identify novel etiologic associations [6,7]. By contrast, extensive small-scale monitoring (or prospective cluster monitoring) avoids many of these problems, in particular the post-hoc bias introduced by finding a cluster in randomness. Therefore, a data and hypothesis driven analysis should be preferred employing the whole spatial and temporal extent of registry data. Additional benefits may be seen in a better use of the full set of cancer registry data which is one major purpose for running cancer registries. Moreover, a monitoring that covers a complete region has advantages in terms of not only screening the putative exposure-associated tumours over time and space but to encompass also other cancer sites which are related to differential spatial distributions. Thus, the spatial incidence patterns of tumours, like breast and prostate cancer, can indicate how screening behaviour varies over space and time. Monitoring can also provide data about the spatial and temporal variation of lifestyle associated tumours that belong to certain risk behaviours (like alcohol or tobacco consumption).

Spatially focussed data may therefore have important implications for public health policies. To conduct comprehensive and extensive spatial risk monitoring programs, various methods have been made available that range from local cluster tests to full Bayesian smoothing methods [5]. Usually, there is no a priori knowledge about the location of “true” clusters in the application of such methods.

This study was planned and conducted with the aim of evaluating the performance of commonly used local cluster tests and Bayesian smoothing methods in terms of their detection and prediction rates when applied in a simulated spatial risk surface. The second aim of the study was to assess the spatial resolution of the methods, that is, to test on which spatial scale clusters are still sufficiently identifiable. The spatial units used were 78 communities and 1983 census tracts. The community level is the lowest spatial unit in the common administrative division of Germany and corresponds to the LAU 2 (level of local administrative units) in the EU. There exist several simulation studies that evaluated the statistical performance of cluster detection tests [8-14] but only few investigated the performance of these tests when using different spatial aggregation level [9,10,15]. Most of these simulation studies were designed for settings with huge sample sizes (10 000-50 000 cases) which cannot be directly compared to the conditions described above where cancer registries deal with much smaller samples sizes and a lower spatial resolution of the administrative data. We aimed to investigate the accuracy and precision of cluster detection tests and Bayesian smoothing techniques when applied to a setting with smaller areas, lower population numbers and fewer cancer cases. For this simulation study a common type of cancer, lung cancer in men and women in the age group between 40 and 79 years, was chosen as sample data.

## Data and methods

### Study area

**Figure 1:**
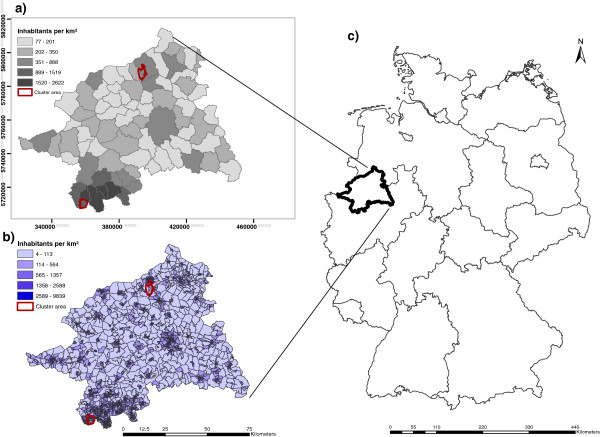
**Overview of the study area (Regierungsbezirk Münster) with the modelled cluster areas.** Two different spatial aggregation scales are shown: **(a)** the 78 communities and **(b)** the 1983 census tracts with the associated population density. Location of the study area in Germany **(c)**.

The study area is located in the northwestern part of Germany (Regierungsbezirk Münster). It consists of 78 communities (Gemeinden), including 4 municipalities (kreisfreie Städte), and corresponds to 1983 census tracts. The mean population density of the Regierungsbezirk is 1533 inhabitants per km^2^, ranging from 4 to 13615 inhabitants per km^2^ between communities (Figure 1). The population data for the 78 communities for the year 2005 and the information on geometric boundaries were obtained from [16]. The population data and the geometric information at census tract-level were purchased from [17]; they were derived from electoral districts with approximately equal size (ca. 500 households).

### Simulation design

For this simulation study, lung cancer (ICD-10: C34) cases occurring in men and women in the age group between 40 and 79 years were chosen as sample data. Spatial cancer risk surfaces were constructed by arbitrarily defining two artificial cluster areas at the level of the census tracts. Within these cluster areas, two magnitudes of risk elevation were applied such that the lung cancer risk was computationally set to be either two- (RR_1_ = 2.0) or four-fold (RR_2_ = 4.0) as high as the observed risk. The two risk areas were nested within larger communities. The northern cancer cluster (encompassing 6 of the total 50 census tracts in that community) had more rural characteristics, that is, a larger area and lower population density. The second cancer cluster was generated in the south (encompassing 37 out of a total of 99 census tracts composing the entire community) with more urban characteristics, that is, a smaller area and units with higher population density (Figure 1).

**Figure 2:**
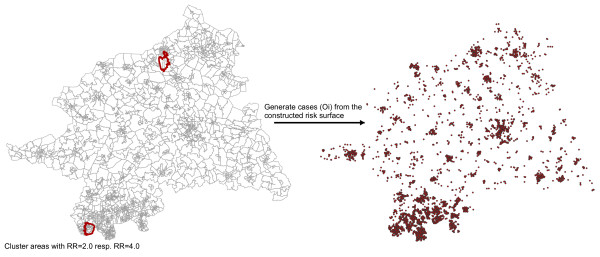
**Overview of the simulation process/design.** In the defined cluster areas relative risks of two resp. four and in the remaining study a relative risk of one were assigned. The geocoded observed cases (O_i_) were generated using an inhomogeneous Poisson process.

**Figure 3:**
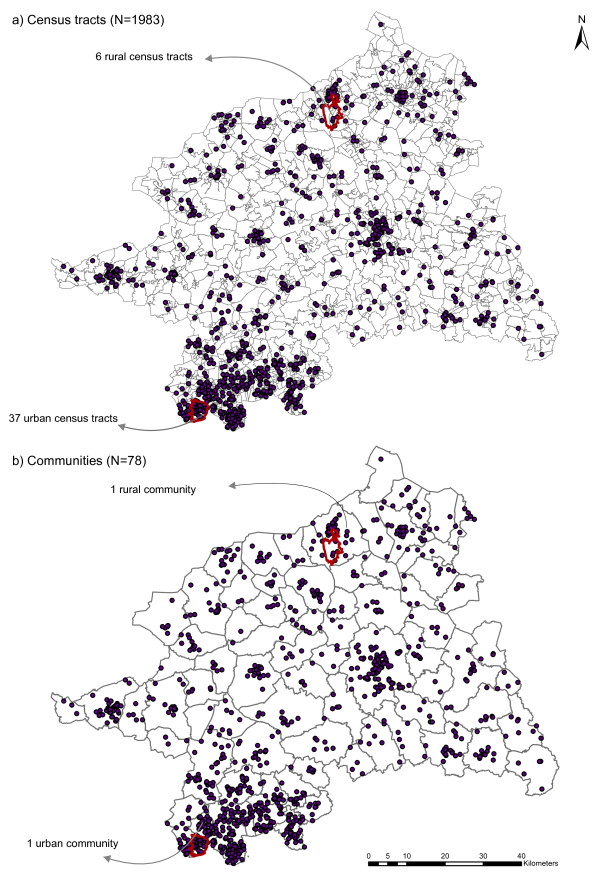
**Spatial aggregation scales of the realised observed cases.** The observed cases (O_i_) were aggregated to the census tracts **(a)**, and to the communities **(b)**.

The expected numbers of cancer cases (E_i_) per census tract were estimated employing the age-standardized incidence rate for lung cancer as obtained from the database of the epidemiological cancer registry of North Rhine-Westphalia [18]. The observed cases (O_i_) were sampled from the four constructed risk surfaces (urban & rural cluster with either RR_1_ = 2.0 or RR_2_ = 4.0) as geocodes using an inhomogeneous Poisson point process (Figure 2). 1000 realisations of the process for each cluster and RR magnitude were generated using function *rpoispp* from the R package spatstat. These realisations (O_i_) were aggregated within census tracts and communities, respectively, and used for the subsequent local cluster tests and Bayesian smoothing methods (Figure 3).

### Local cluster tests

Local cluster tests aim to provide information about the spatial location of suspected clusters. The statistical concept behind the local cluster tests rests on the assumption that disease risk is constant across the study population (constant risk hypothesis or null hypothesis, implying identical risk for each individual). The standardized incidence ratio (SIR), defined as ratio of observed to expected cases, is commonly used as a measure of relative disease risk. A constant risk implies that SIR = 1.0. A SIR value that is significantly larger than 1 indicates a disease cluster. Two types of local cluster tests were applied: The first is based on local measurements of spatial autocorrelation (local Moran’s I) and the second is based on variously defined windows that scan the study region for elevated disease risk (Kulldorff spatial scan statistic; Besag & Newell) [19]. We applied the methods provided in the R packages DCluster (version 0.2-2) [20] and spdep [21]. For local Moran’s I, Kulldorff spatial scan statistic, and the method of Besag & Newell [20], all computations were performed with R version (2.13.1) [22].

#### - Local Moran’s I

The local Moran’s I measures the deviations of a value in comparison to the mean of the neighbouring areas. In this study, the standardized residuals, as defined in [5], were used. At census tract level, a significantly positive statistic of I (p-value ≤ 0.05) was used in order to detect adjacent census tracts of high risk (hot spot clustering). By contrast, at community level, significantly negative values of I (p-value ≤0.05) were considered because here the aim is detection of communities that deviate extremely from neighbouring communities (local outliers). The R-function *localmoran* from R package spdep [21] was used under the assumption of normality and through the randomisations approach [5]. Due to the small number of spatial neighbours at community level, the exact (*localmoran.exact*) form of the standard deviates were calculated because the assumption of the normal distribution potentially lead to errors of inference [23,24]. The p-values were adjusted for multiple testing using the false discovery rate (FDR) [25]. This criterion controls the expected proportion of false discoveries among the rejected hypotheses and has been found to be more powerful in the detection of spatial clusters than the family-wise error rates [26]. The FDR approach is implemented in the R-function *p.adjustSP* from the package spdep [21] which additionally adjusts by accounting for spatial neighbours: the p-values are based on the number of neighbours (+1) of each region, rather than the total number of regions.

#### - Kulldorff’s spatial scan statistic

The Kulldorff spatial scan statistic [27] is based on the likelihood ratio statistics. In this approach, a variable circular scan window was applied to the study area, with radius increasing up to 50% of the population at risk. The actual likelihood ratio is calculated for each circle as the ratio of observed to expected cases within and outside the scan window (L_actual_). The likelihood function assuming Poisson distributed cases is proportional to:

(1) $${\left(\frac{c}{E\left[c\right]}\right)}^{c}{\left(\frac{C-c}{C-E\left[c\right]}\right)}^{C-c}I\left(c>E\left[c\right]\right)$$

where *c* is the number of cases, *E*[*c*] is the expected number of cases within a circle and (C-c) and (C-E(c)) the observed and expected cases outside the scan window. We chose the indicator function to be 1.0 if the observed number of cases was higher than expected. Under the null hypothesis, assuming a constant risk over the study area, datasets are generated and the maximum likelihood ratio (L_0_) is saved. The statistical significance is computed by means of Monte-Carlo simulation and yields the probability that L_actual_ is exceeded anywhere in the study area; clusters least consistent with the null hypothesis are highlighted. The Kulldorff spatial scan statistic adjusted for the multiple testing by the use of one test only. The analyses were conducted with function *opgam* from the R package DCluster [20], the significance was defined at the 0.05 level and the p-values were calculated using 9999 Monte-Carlo realizations. The most likely clusters were considered with a p-value ≤ 0.05.

#### - Approach of Besag & Newell

In the method of Besag & Newell [28] the scan window is defined by the number of enclosed cases (k). In the case of rare diseases, like cancer, the number of enclosed cases varies between 2 and 10 [19]. This approach evaluates the probability whether the specified k cases are observed in fewer regions [5]. To this end, the actual number of regions (l_i_) is compared with the number of regions under the constant risk hypothesis (L_i_) using a Poisson distribution (1-Pr(L_i_ ≤ l_i_) ~ Poisson(E_i_)). The analyses were made with the R function *opgam* R package DCluster [20] and the p-values were adjusted using the FDR approach implemented in the R-function *p.adjust* from the package stats [29]. The number of k enclosed cases was arbitrarily chosen and we used k_CT_ = 5, k_CT_ = 10, k_CT_ = 13 for census tracts and k_com_ = 15, k_com_ = 20, and k_com_ = 30 for communities.

### Bayesian smoothing methods

Smoothing methods do not primarily detect clusters. Their aim is to model/estimate the spatial distribution of the true underlying disease risk because mapping the crude SIR has major drawbacks, especially the instability of the estimates in region with low background population. Smoothing methods therefore try to remove the random noise caused by the unstable estimates. It is also possible to deploy these smoothing methods in the field of cancer surveillance with the aim to identify risk areas.

#### - Empirical Bayes smoothing

The Bayesian smoothing methods define the risk measure as a random variable and therefore assign a distribution to the estimate of the “true” risk (= theta(θ_i_)). In the empirical procedures, the parameters defining this risk distribution (= priors) are estimated from the data. The estimates of theta were stabilized through borrowing information from the prior mean. The amount of strength borrowed depends on the stability of the crude local SIR (or risk measure) as measured by the prior variance [5].

Three models were applied: two global (non-spatial) models (Poisson-Gamma (PG) model and log-normal model) with smoothing the risk estimates towards the global mean, and a local (spatial) model that smoothes the risk to a spatial neighbourhood mean. Both global models were implemented in the DCluster R package [20] and the local model in the spdep package [21]. The PG-model assumes that the observed cases (O_i_) are Poisson distributed and because it is likely that the counts (O_i_) are overdispersed, it is reasonable to define theta as Gamma distributed with θ_i_ ~ Gamma(α,β). The priors α (mean) and β (variance) were estimated using the EM-algorithm from [30]. The R-function used was *empbaysmooth* from R package DCluster [20]. In the log-normal model, the SIR is estimated as the logarithm of theta assuming a normal distribution with common mean (α) and variance (β) [31]. These priors are also estimated using the EM-algorithm proposed by Clayton & Kaldor [30]. This model is implemented in the DCluster package [21] under the function *lognormalEB*. In the local EB model (Marshall 1991) the crude risk estimate is shrunk toward a local (neighbourhood) mean. The EB estimator of Marshall (1991) assumes no prior distribution of the risk estimates and is therefore based only on their prior mean (α) and variance (β). The local EB estimator is implemented in the R-function *EBlocal* from the package spdep [21]. The spatial neighbourbood definition is based on the rook contiguity where a spatial neighbour shares at least a common border.

#### - Hierarchical Bayes smoothing (BYM model)

In hierarchical Bayes methods, the parameters describing the distribution of theta_i_ are not estimated from data but are further specified through hyperpriors. The hyperpriors describe the distribution of the priors and are estimated by means of MCMC-simulations. These are used to derive the posterior distribution of theta_i_. The BYM-model [32] split the variation of the theta_i_ into two components: a correlated random term (u_i_) that depends on values from the neighbourhood (= correlated heterogeneity), and an uncorrelated random component (v_i_) which describes the heterogeneity (= uncorrelated heterogeneity) in the study area. The BYM model was implemented in the WinBUGS software using MCMC methods, in particular Gibbs sampling [33]. A burn-in of 20 000 iterations was performed and the posterior distribution was obtained using a sample of 10 000 iterations. The point estimates of theta from the four Bayesian models were used in the subsequent (cluster) evaluations.

### Evaluation of the simulation results

There were two simulated cluster communities out of a total of 78 communities and 43 artificial cluster tracts at the level of the 1983 census tracts. The accuracy of the local cluster tests was assessed by cross-classifying the ‘true’ reference status in the simulated risk surface with the results of the different cluster tests. The categorization was dichotomous, that is, we distinguished only cluster and non-cluster. Based on the cross-classifications, we obtained numbers of correctly detected clusters (True Positives, TP), falsely detected clusters (False Positives, FP), non-detected clusters (False Negatives, FN) and correctly classified non-clusters (True Negatives, TN) as means (census tracts) and as sums (communities) over 1000 realizations. We calculated the detection rate (= true positive rate) as DR = TP/(TP + FN)) and the specificity as Sp = TN/(FP + TN) for each cluster test. Furthermore, the positive predictive value was calculated as PPV = TP/(TP + FP) and the likelihood ratio of a positive test as LR + = (TP/(TP + FN)/ (FP/(FP + TN), with the PPV providing information about the probability that a positive test result correctly predicts a true cluster, and the LR + describing how many times more likely a positive test result is in a cluster area compared to non-cluster areas. The described measures are presented with 95% confidence intervals (CI) at census tract level.

The statistical power, that is, the probability of accepting the null hypothesis of a constant risk over the study area although it is not true, was assessed for the local cluster tests and for each of the eight dataset combinations (2 cluster sites × 2 risk magnitudes × 2 gender groups). We use an approximate approach, because local Moran’s I provides no global statistic. The approximate power of rejecting the null hypothesis (no clustering) was calculated as proportion of at least one minimum p-values ≤ 0.05 over 1000 realizations of each dataset combination. The results of the Bayesian smoothing methods were assessed using the Receiver Operating Characteristics (ROC) curves since they do not require a specific cut-off-value of the risk estimate for defining a cluster. The ROC curves plot the false positive rate versus the detection rate. For each cluster site, risk magnitude, gender and data aggregation level a ROC curve is presented for the four Bayesian methods averaged over the 1000 realizations.

## Results

### Results of the simulation process

The results of the eight (2 cluster types × 2 risk magnitudes × 2 gender groups) times 1 000 risk realizations are displayed in Table 1 which contains the mean of the expected counts based on the background incidence, the observed sampled counts based on the artificial risk surface, and the simulated relative risk increases (expressed as SIR). On the census tract scale, an effective realization of the two-and four-fold risk increases was achieved on average for the urban and rural clusters in men and women. However, the 95% Poisson CIs were much narrower in the urban clusters while they clearly included the null value for a RR_1_ = 2.0 in the rural clusters. The mean SIR values for the non-cluster areas were 1.0 with a narrow 95% CI. On the community scale, the SIR values were much more weakly elevated: the point estimates ranged from 1.06 to 1.35 with RR_1_ = 2.0 and from 1.25 to 2.07 for a RR_2_ = 4.0. At this scale, only the urban clusters with a simulated RR_2_ = 4.0 and the male urban cluster for RR_1_ = 2.0 showed 95% CIs for the SIR that did not include the null value. On the other hand, the CIs were wide in the rural clusters and they included mostly the null value. The average SIR was 1.0 in the non-cluster areas with narrow CIs.

**Table 1:** Summary results of 1000 realizations from an inhomogeneous Poisson process

**Lung cancer cases**	**Cluster**	**Expected**^**1 **^**(mean)**	**Observed (mean) RR = 2.0**^**2**^	**SIR (mean) RR = 2.0**	**SIR 95% Poisson CI**^**3 **^**RR = 2.0**	**Observed (mean) RR = 4.0**^**2**^	**SIR (mean) RR = 4.0**	**SIR 95% Poisson CI**^**3**^**RR = 4.0**
**Census tracts level**								
**Males**	Urban cluster	18	35	1.92	1.38-2.67	69	3.78	3.03-4.85
**Females**	Urban cluster	6	13	2.13	1.26-3.72	25	4.24	2.27-7.04
**Males**	Rural cluster	3	6	2.08	0.90-4.45	12	4.11	2.82-6.17
**Females**	Rural cluster	1	2	2.2	0.50-7.99	4	4.32	1.5-10.66
**Males**	No cluster, urban	1000	996	1	0.94-1.06	998	1	0.94-1.10
	No cluster, rural		1004		0.94-1.06	1010	1	0.95-1.07
**Females**	No cluster, urban	358	369	1	0.94-1.06	358	1	0.9-1.1
	No cluster, rural		371	1.03	0.94-1.15	363	1	0.91-1.12
**Community level**								
**Males**	Urban cluster	49	66	1.35	1.10-1.71	99	2.03	1.66-2.46
**Females**	Urban cluster	18	25	1.36	0.94-2.10	37	2.07	1.49-2.84
**Males**	Rural cluster	30	33	1.11	0.78-1.55	39	1.32	0.95-1.78
**Females**	Rural cluster	11	12	1.06	0.62-1.92	13	1.25	0.69-2.04
**Males**	No cluster, urban	942	934	1	0.93-1.06	966	1.03	0.99-1.13
	No cluster, rural		967	1.03	0.96-1.09	983	1.04	0.98-1.11
**Females**	No cluster, urban	336	333	1	0.9-1.1	346	1.03	0.93-1.14
	No cluster, rural		346	1.03	0.93-1.1	354	1.05	0.95-1.17

CI = confidence interval of 1000 realizations.

^1^Expected under the null hypothesis (= background incidence).

^2^Observed with sampling using an inhomogeneous Poisson process.

^3^Boice-Monson Method.

### Results of the local cluster tests

The statistical power of the Kulldorff spatial scan statistic, the approach of Besag & Newell (BN) and local Moran’s I (LMI) is given in Table 2. The results show at the census tract scale that all tests have a sufficient power (100%) to detect clustering under the eight risk (dataset) combinations. At community scale the power is generally decreased, but while the Kulldorff spatial scan statistic and the LMI still had statistical power (>63%) to detect clustering, the BN method showed a considerable loss in power. In fact, only the female urban cluster realization with a four-fold risk increase could be identified with 90% power for 30 enclosed cases (k = 30).

**Table 2:** Power of the Kulldorff spatial scan statistic, the Besag & Newell statistic, and the local Moran’s I statistic for detecting spatial clustering

			**Census tracts**					**Communities**				
			**Besag & Newell**			**Kulldorff spatial scan statistic**	**Local Moran’s I**	**Besag & Newell**			**Kulldorff spatial scan statistic**	**Local Moran’s I**
			**k = 5**	**k = 10**	**k = 13**			**k = 15**	**k = 20**	**k = 30**		
**RR = 2.0**	**urban**	**Males**	1.0	1.0	1.0	1.0	1.0	0.21	0.22	0.23	0.86	0.78
		**Females**	1.0	1.0	1.0	1.0	1.0	0.25	0.39	0.42	0.76	0.84
	**rural**	**Males**	1.0	1.0	1.0	1.0	1.0	0.19	0.21	0.22	0.75	0.82
		**Females**	1.0	1.0	1.0	1.0	1.0	0.22	0.4	0.35	0.73	0.82
**RR = 4.0**	**urban**	**Males**	1.0	1.0	1.0	1.0	1.0	0.22	0.23	0.26	1.0	0.63
		**Females**	1.0	1.0	1.0	1.0	1.0	0.25	0.36	0.9	0.98	0.78
	**rural**	**Males**	1.0	1.0	1.0	1.0	1.0	0.19	0.21	0.25	0.8	0.83
		**Females**	1.0	1.0	1.0	1.0	1.0	0.23	0.49	0.48	0.74	0.83

The accuracy of the cluster locations at census tract level using the eight different model realizations are displayed in Table 3 for male and in Table 4 for female lung cancer cases. All local cluster tests showed a very high specificity reflecting the large number of non-cluster areas. For all local cluster tests there is an increase in the mean detection rate (DR) with increasing risk magnitudes in the cluster areas. The increase of the mean DR is especially distinct in the urban cluster. Kulldorff spatial scan statistic had the highest detection rate in the urban cluster regardless of the risk magnitude but it also produced the highest number of false positives which resulted in low values for PPV and LR+. With a cluster RR_1_ = 2.0, the Besag & Newell test for the urban cluster had only mean DRs lower than 0.5 while the positive predictive power (PPV) was in the range of Kulldorff spatial scan statistic for that risk. With cluster RR_2_ = 4.0, the mean DR for the BN rose above 0.9 with PPVs between 0.27 and 0.48, whereas Kulldorff spatial scan statistic had only a mean PPV 0.09. The local Moran’s I showed the weakest ability of all applied local cluster tests to detect and predict clusters with RR_1_ = 2.0 but it had the highest mean PPV (0.51) for the urban clusters of lung cancer in males when RR_2_ = 4.0. Further, it was the only method where the mean DR increased with simultaneously decreasing of FPs when the RR was higher. However, the test accuracy in women was generally lower than in men. Of note, in the rural clusters of lung cancer, the DR, PPV and LR + were all consistently very low, both in men and women and regardless of the risk magnitude.

**Table 3:** Summary of the local cluster test results for male lung cancer for both risk magnitudes, by census tract level

				**Urban cluster**							**Rural cluster**						
	**Test**		**Parameter**	**TP**	**FP**	**FN**	**DR**	**Sp**	**PPV**	**LR+**	**TP**	**FP**	**FN**	**DR**	**Sp**	**PPV**	**LR+**
**RR = 2**	**Besag & Newell**	**k = 5**	**Mean**	4	15	33	0.11	0.99	0.08	16.6	1	16	5	0.11	0.99	0.02	18.0
			**CI [95%]**	0-16	2-24	20-36	0-0.43	0.98-0.99	0-0.32	0-131.5	0-4	4-29	2-6	0-0.67	0.98-1	0-0.13	0-131.8
		**k = 10**	**Mean**	12	32	25	0.33	0.98	0.28	24.0	1	32	5	0.10	0.98	0.02	6.8
			**CI [95%]**	2-37	7-48	0-35	0.054-1	0.97-0.99	0.06-0.74	3.3-149	1-0	15-58	5-6	0-0.67	0.97-0.99	0-0.13	0-49.4
		**k = 13**	**Mean**	15	39	22	0.41	0.98	0.29	25.4	1	38	5	0.09	0.98	0.01	5.5
			**CI [95%]**	2-37	6-60	0-34	0.054-1.24	0.96-0.99	0.06-0.77	3.5-181.7	3-1	17-62	3-5	0-0.67	0.96-0.99	0-0.11	0-41.1
	**Kulldorff spatial scan**		**Mean**	30	135	7	0.82	0.93	0.27	24.6	0	98	6	0.03	0.95	0.00	1.0
	**statistic**		**CI [95%]**	2-37	11-588	0-33	0.05-1	0.64-0.99	0.034-0.63	1.8-90.6	0-1	14-425	5-6	0-0.03	0.7-0.99	0-0.03	0-1.5
	**Local Moran’s I**		**Mean**	6	30	31	0.17	0.98	0.17	12.1	0	34	6	0.00	0.98	0.00	0.0
			**CI [95%]**	0-15	18-43	22-37	0-0.41	0.97-1	0-0.38	0-33	0-0	21-48	6-6	0-0	0.98-1	0-0	0-0
**RR=4**	**Besag & Newell**	**k = 5**	**Mean**	22	16	15	0.59	0.99	0.27	86.6	3	16	3	0.55	0.99	0.08	82.0
			**CI [95%]**	12-34	4-26	3-24	0.32-0.92	0.98-0.99	0.14-0.48	35.9-305	0-6	5-30	0-6	0-0.16	0.99-1.0	0-0.2	0-0
		**k = 10**	**Mean**	34	39	3	0.92	0.98	0.48	51.7	2.781	35	3	0.46	0.98	0.08	29.2
			**CI [95%]**	29-37	12-66	0-7	0.78-1	0.96-0.99	0.34-0.72	26.3-133.5	0-6	11-63	0-6	0-0.16	0.98-1	0-0.19	0-105.2
		**k = 13**	**Mean**	35	49	2	0.95	0.97	0.44	43.3	3	42	3	0.43	0.98	0.06	22.8
			**CI [95%]**	32-37	16-86	7-10	0.86-1	0.95-0.99	0.28-0.66	21.2-102.4	0-6	12-82	0-6	0-0.16	0.97-1.0	0-0.17	0-105.2
	**Kulldorff spatial scan**		**Mean**	37	475	0	1.00	0.76	0.09	5.7	1	136	5	0.12	0.93	0.01	2.8
	**statistic**		**CI [95%]**	37-37	103-914	0-0	1-1	0.53-0.94	0.04-0.25	2.1-17.7	0-1	18-460	5-6	0-0.027	0.7-0.98	0-0.036	0-2
	**Local Moran I**		**Mean**	24	24	13	0.65	0.99	0.51	57.7	0	35	6	0.00	0.98	0.00	0.0
			**CI [95%]**	15-31	13-35	6-22	0.41-0.84	0.98-0.99	0.34-0.68	27.1-109.2	0-0	21-48	6-6	0-0	0.96-0.99	0-0	0-0

**Table 4:** Summary of the local cluster test results for female lung cancer for both risk magnitudes, by census tract level

				**Urban cluster**							**Rural cluster**						
	**Test**		**Parameter**	**TP**	**FP**	**FN**	**DR**	**Sp**	**PPV**	**LR+**	**TP**	**FP**	**FN**	**DR**	**Sp**	**PPV**	**LR+**
**RR = 2**	**Besag & Newell**	**k = 5**	**Mean**	5	24	32	0.14	0.99	0.09	13.41	0	23	6	0.04	0.99	0.01	5.12
			**CI [95%]**	0-19	6-50	18-37	0-0.51	0.97-1	0-0.33	0-66.4	0-3	6-50	3-6	0-0.5	0.97-1	0-0.08	0-43
		**k = 10**	**Mean**	10	42	27	0.27	0.98	0.19	16.36	0	40	6	0.05	0.98	0.01	3.44
			**CI [95%]**	0-31	6-99	5-37	0-0.86	0.94-1	0-0.6	0-74.7	0-4	3-114	2-6	0-0.67	0.94-1	0-0.08	0-25
		**k = 13**	**Mean**	12	49	25	0.32	0.97	0.18	16.55	0	45	6	0.05	0.98	0.01	3.65
			**CI [95%]**	0-35	6-120	2-37	0-0.95	0.94-1	0-0.51	0-71	0-4	3-114	2-6	0-0.67	0.94-1	0-0.07	0-24.7
	**Kulldorff spatial scan**		**Mean**	14	117	23	0.38	0.94	0.14	10.3	1	98	6	0.14	0.95	0.01	4.6
	**statistic**		**CI [95%]**	0-37	13-625	0-37	0-1	0.62-1	0-0.47	0-46.2	0-6	11-429	5-6	0-1	0.78-1	0-0.12	0-44.9
	**Local Moran’s I**		**Mean**	4	38	33	0.10	0.98	0.09	5.64	0	41	6	0.00	0.99	0	0
			**CI [95%]**	0-10	24-51	27-33	0-0.27	0.97-0.99	0-0.25	0-17.5	0-0	26-55	6-6	0-0	0.97-0.99	0-0	0-0
**RR = 4**	**Besag & Newell**	**k = 5**	**Mean**	23	26	14	0.61	0.99	0.23	56.27	1	25	5	0.17	0.99	0.02	15.04
			**CI [95%]**	7-34	8-53	3-30	0.19-0.92	0.97-1	0.08-0.42	10.7-163	0-4	7-53	2-6	0-0.67	0.97-1	0-0.15	0-82.4
		**k = 10**	**Mean**	32	53	5	0.87	0.97	0.41	40.92	1	43	5	0.176	0.98	0.02	8.61
			**CI [95%]**	16-37	13-113	0-20	0.46-1	0.94-1	0.2-0.7	14.2-122.7	0-5	7-105	1-6	0-0.83	0.95-1	0-0.13	0-61.8
		**k = 13**	**Mean**	34	63	3	0.93	0.97	0.37	33.86	2	52	5	0.16	0.97	0.02	5.6
			**CI [95%]**	18-37	17-141	0-18	0.51-1	0.93-0.99	0.19-0.65	11.8-98.7	0-6	5-133	0-6	0-0.83	0.93-1	0-0.1	0-36.6
	**Kulldorff spatial scan**		**Mean**	37	264	0	0.99	0.86	0.19	13.6	0	109	6	0.06	0.94	0.00	1.6
	**statistic**		**CI [95%]**	31-37	36-873	0-5	0.84-1	0.55-0.98	0.04-0.45	2.2-43.2	0-1	14-441	5-6	0-0.17	0.72-0.99	0-0.03	0-11.4
	**Local Moran’s I**		**Mean**	13	34	24	0.35	0.98	0.28	21.54	0	40	6	0.00	0.98	0.00	0.0
			**CI [95%]**	4-21	20-49	16-33	0.11-0.57	0.97-0.99	0.11-0.45	6.2-43.5	0-0	27-54	6-6	0-0	0.97-0.99	0-0	0-0

**Table 5:** Summary of the results for male and female lung cancer, by community level

				**Male lung cancers**				**Female lung cancers**			
**RR**	**Method**		**Parameter**	**TP urban**	**FP urban**	**TP rural**	**FP rural**	**TP urban**	**FP urban**	**TP rural**	**FP rural**
**RR = 2.0**	**Besag &**	**k = 15**	**sum**	210	80	1	269	27	17	1	361
	**Newell**		**DR**	0.21		0.00		0.03		0.00	
			**PPV**	0.72		0.00		0.61		0.00	
			**LR+**	202.13				135.9		0.00	
		**k = 20**	**sum**	220	40	240	92	39	51	414	384
			**DR**	0.22		0.24		0.04		0.41	
			**PPV**	0.85		0.72		0.43		0.52	
			**LR+**	423.50		200.87		60.4		69.45	
		**k = 30**	**sum**	220	150	1	361	50	57	1	842
			**DR**	0.22		0.00		0.05		0.00	
			**PPV**	0.59		0.00		0.47		0.00	
			**LR+**	112.93		0.00		67.5		0.00	
	**Kulldorff**		**sum**	501	3974	92	3770	744	3220	80	3996
	**spatial scan**		**DR**	0.50		0.09		0.74		0.08	
	**statistic**		**PPV**	0.11		0.02		0.19		0.02	
			**LR+**	9.71		1.88		17.7		1.54	
	**Local**		**sum**	190	1512	37	1728	34	126	52	1569
	**Moran’s I**		**DR**	0.19		0.04		0.03		0.05	
	**exact**		**PPV**	0.11		0.02		0.21		0.03	
			**LR+**	9.68		1.65		20.78		2.55	
**RR = 4.0**	**Besag &**	**k = 15**	**sum**	126	172	2	260	242	130	1	368
	**Newell**		**DR**	0.13		0.00		0.24		0.00	
			**PPV**	0.42		0.01		0.65		0.00	
			**LR+**	56.41		0.59		143.34		0.00	
		**k = 20**	**sum**	232	102	224	120	384	382	464	610
			**DR**	0.23		0.22		0.38		0.46	
			**PPV**	0.69		0.65		0.50		0.43	
			**LR+**	175.14		143.73		77.40		58.57	
		**k = 30**	**sum**	250	80	2	321	920	810	2	1105
			**DR**	0.25		0.00		0.92		0.00	
			**PPV**	0.76		0.01		0.53		0.00	
			**LR+**	240.63		0.48		87.46		0.00	
	**Kulldorff ’s**		**sum**	999	11542	351	5590	916	6506	155	4469
	**spatial scan**		**DR**	1.00		0.35		0.92		0.16	
	**statistic**		**PPV**	0.08		0.06		0.12		0.03	
			**LR+**	6.66		4.83		10.84		2.67	
	**Local**		**sum**	398	732	106	1636	304	1130	72	1588
	**Moran’s I**		**DR**	0.40		0.11		0.30		0.07	
	**exact**		**PPV**	0.35		0.06		0.21		0.04	
			**LR+**	41.87		4.99		20.72		3.49	

TP = true positive, FP = false positive; DR = detection rate, PPV = positive predictive value.

LR + = likelihood ratio of a positive test result.

At the community level, a high number of non-cluster communities (n = 76) was compared in each dataset combination to only one community that harboured the cluster areas in its borders (Table 5). Generally, however, the same pattern can be observed as at census tract level: The urban clusters are better detected than the rural ones and the clusters were better detected in the male population than in the female. The overall DR for the Kulldorff spatial scan statistic and LMI increased with higher simulated cluster RR and the Kulldorff spatial scan statistic had the highest DRs, but at the cost of an immense number of FPs. The DRs using the BN test were similar for the two cluster RR magnitudes but the FPs were higher when the RR was higher. Only the LMI showed that increases of the DR were accompanied by remarkable FP decreases.

### Results of the Bayesian smoothing methods

**Figure 4:**
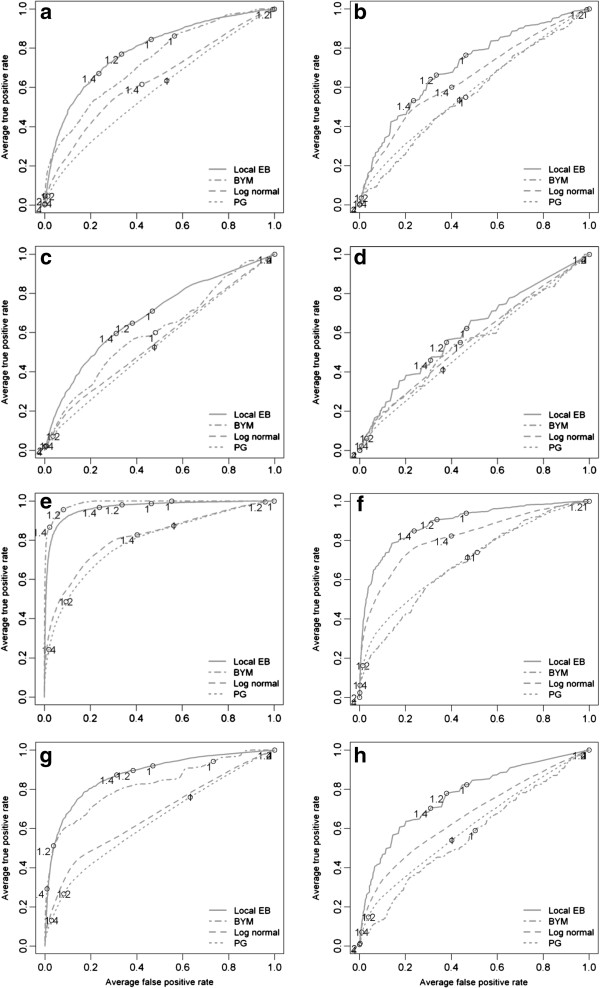
**Averaged ROC curves of the four applied Bayesian smoothing models at census tract level.** The letter indicating the different risk realizations: **(a)** urban cluster in the male population (RR = 2.0); **(b)** rural cluster the male population (RR = 2.0); **(c)** urban cluster in the female population (RR = 2.0); **(d)** rural cluster the male population (RR = 2.0); **(e)** urban cluster in the male population (RR = 4.0); **(f)** rural cluster in the male population (RR = 4.0); and **(g)** rural cluster in the female population (RR = 4.0).

**Figure 5:**
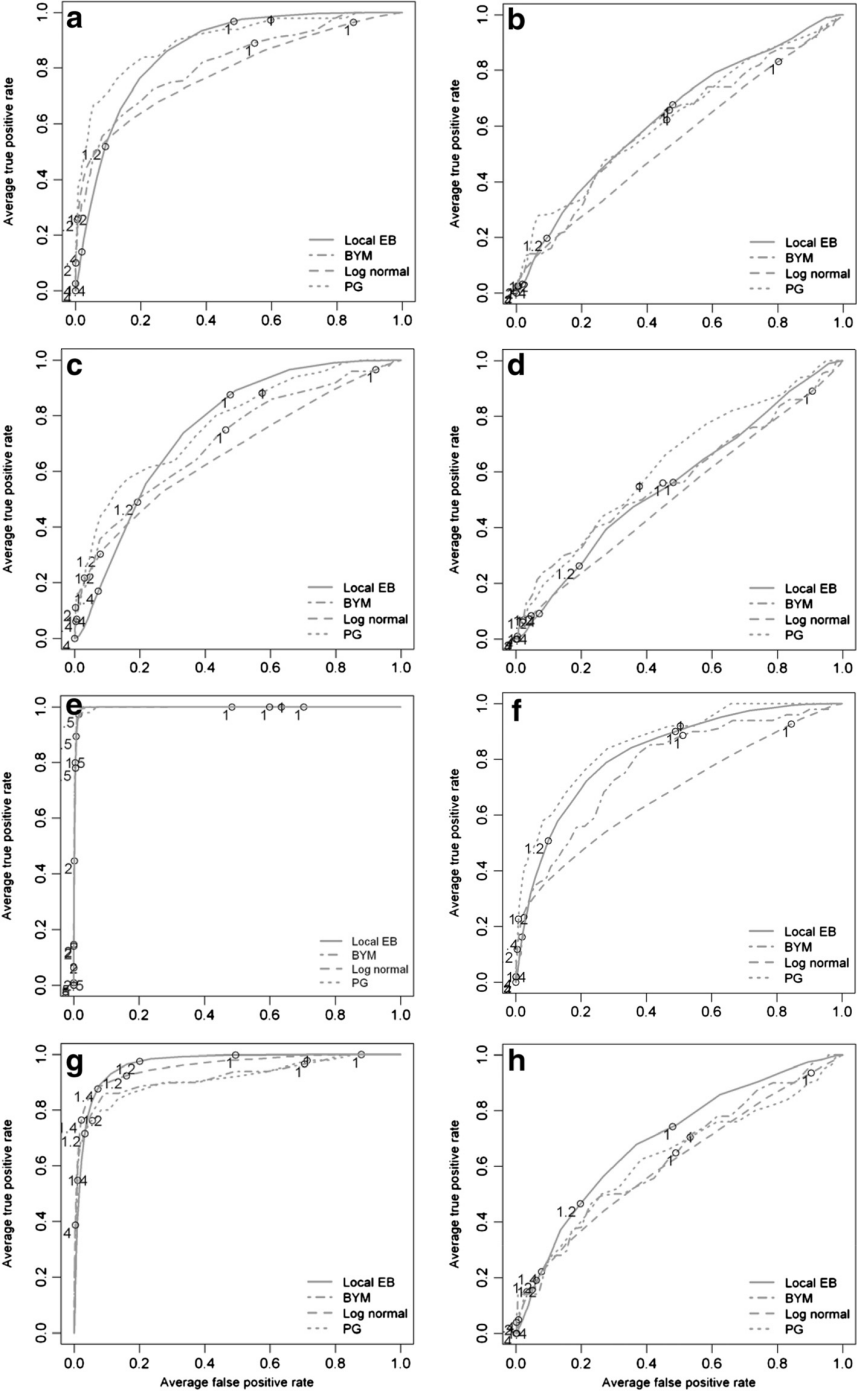
**Averaged ROC curves of the four applied Bayesian smoothing models at community level.** The letter indicating the different risk realizations: **(a)** urban cluster in the male population (RR = 2.0); **(b)** rural cluster the male population (RR = 2.0); **(c)** urban cluster in the female population (RR = 2.0); **(d)** rural cluster the male population (RR = 2.0); **(e)** urban cluster in the male population (RR = 4.0); **(f)** rural cluster in the male population (RR = 4.0); and **(g)** rural cluster in the female population (RR = 4.0).

The results of the Bayesian smoothing techniques are summarized using ROC curves in Figure 4 (census tracts) and Figure 5 (communities). Across all cluster types and cluster risk magnitudes, the methods that implement a spatial neighbourhood, and therefore smooth the risk estimate towards a local mean, performed better than the global methods. At census tract scale and with a cluster RR_1_ = 2.0, the local EB method had the highest mean DR (between 0.6 and 0.7) with the lowest average FP rate at a threshold of 1.4 in the urban clusters. For the rural cluster the threshold was the same but the mean DR was lower (0.5-0.6) with a higher mean FPR. In the female population, the ROC curves are close to the diagonal line, denoting that the methods have only a minor (urban cluster) or no (rural cluster) discriminatory power. With increasing cluster risk magnitude the test accuracy for all methods was improved, that is, the area under the curve (AUC) was augmented. For the urban cluster in men (Figure 4e), the BYM model showed a slightly better performance than the local EB: the BYM model achieved its highest mean DR (>0.8) with a minimum FPR (<0.05) at threshold values between 1.2 and 1.4 while the local EB had at comparable threshold values a higher FPR (>0.2). For the urban cluster in women (Figure 4g), the same pattern was observed: For same risk threshold of 1.2 the local EB model had a higher DR (>0.8) but with a higher mean FPR (>0.20), while the BYM model had a lower mean DR (≈0.5) but with a lower FPR (<0.05).

In general, similar patterns were observed at the community level. The local EB achieved the highest accuracy among all smoothing methods and test accuracy increased with increasing cluster risk magnitude. However, for RR_1_ = 2.0, the mean DR were lower and the mean FPR higher as at census tract level. With increasing cluster RR magnitude the mean DR for the urban clusters (Figure 5e and f) increased to almost 1.0 with a mean FP <0.05 at thresholds between 1.2 and 1.4.

## Discussion

The aim of this simulation study was to evaluate different methods in their ability to identify spatial clusters of lung cancer using real-life data from an epidemiological cancer registry in Germany. Little is known about the performance of local cluster tests and Bayesian smoothing methods under conditions that differ by relative risks and spatial scale, that is, small and large population sizes and the respective data aggregations. We found that the local Bayesian smoothing models (local EB and BYM) generally had a better test accuracy than the global models. However, at census tract level and for a RR = 2.0, the local clusters tests generally showed lower FPRs than the Bayesian smoothing methods but with comparable DRs. Also when increasing the cluster RR magnitude, the local cluster tests had lower FPRs with comparable the DRs. Only at the community level and for a four-fold risk magnitude this pattern was reversed: with comparable DR the smoothing models had lower FPRs.

We implemented a simulation process with eight different conditions under which the method performance was evaluated. The conditions encompassed the comparisons of two magnitudes of cluster risk elevations (RR_1_ = 2.0 and RR_2_ = 4.0), of small scale (census tracts) and large scale (communities) population samples and, on each scale, that of densely (urban) with that of sparsely (rural) populated areas. At the census tract level, there was high agreement between the risk increments realised in the simulation and the underlying RR, that is, the realised relative risks were RR ≈ 2.0 resp. RR ≈ 4.0 for both, the urban and the rural clusters. This came, however, with a loss in precision, that is, wider confidence intervals, in the rural clusters: smaller observed and expected counts lead to higher variances of the SIR, a phenomenon known as the ‘small number problem’ or SNP. By contrast, the risk realisations at the community level were affected by a dilution effect because the higher aggregation at this spatial scale tends to markedly attenuate the two-to four-fold risk increases that were present in only a fraction of all the areas that constituted the community. Of note, only 36% of the urban community population was actually affected by the risk increase and only 10% in the rural cluster community. Therefore, given aggregated large scale conditions, risk elevations that are present in only a fraction of the total population result in lower total risk elevations at the aggregated level such that cluster detectability is a priori always reduced. Likewise, differences in the precision of the realised risk between the male and female populations could also be attributed to different numbers given that lung cancers in males are about three times as frequent as in females. Thus, urban clusters at census tract level in men represented in our study the most favourable condition for the test methods to perform, whereas rural cluster at community level in women reflected the most adverse condition. In summary, the described conditions affect the ability of the tests to detect cancer clusters and need to be considered appropriately when interpreting test results.

### Local cluster tests

At census tract level, all cluster tests showed a power of 100% for the eight simulation scenarios, probably because the risk was realized in a sufficient manner. The statistical power is primarily influenced by two factors: the sample size and the true difference between the null and alternative hypothesis [34]. Therefore, the subsequent analysis of the location accuracy of the tests is not affected by low power. By contrast, on the community level a decrease in power was observed that was most likely due to the dilution of the realized risk as a consequence of data aggregation and the different samples sizes (male vs female population, urban versus rural population density). The fact, that the LMI showed the lowest power for the most favourable cluster scenario, e.g. highest risk realization und highest sample size (urban cluster in male & female population with a RR_2_ = 4.0) was probably due to a less production of false positive locations than in the other cluster scenarios. In addition, it became also apparent that the power of the BN method is very sensitive to the choice of k. Regarding the accuracy of location, Kulldorff’s spatial scan statistic had the greatest ability among the local cluster tests to correctly identify lung cancer clusters in urban as well as rural environments; this was particularly true at the census tract level. However, the predictive power, that is, the probability that a positive test result correctly represented a cluster, was at the same time low due to the high numbers of FPs. These results are consistent with the findings of Aamondt et al. [35] who applied a comparable simulation design in order to evaluate the sensitivity and specificity of three local cluster tests (Kulldorff spatial scan statistic, BYM, GAM) in Norwegian municipalities (comparable in area and population sizes to German communities). They found an average detection rate for urban clusters of 75% when simulating a risk increase of 50% (RR = 1.5) and of 80% for a RR = 4.0. For a comparable rural cluster they reported a detection rate of 51% (RR = 1.5) and of 87% (RR = 4), respectively. The higher DR values for the rural cluster in [35] were attributable to a larger sample size, that is, 1.1% of the total Norwegian population was included in this cluster. Unfortunately, Aamondt et al. [35] provide no information about the numbers of the FPs and therefore about the predictive abilities of the applied tests. Huang et al. [36] showed in their simulation study that Kulldorff’s spatial scan statistic achieved only a PPV of 0% for a RR of 1.2 for lung cancer in male and female with a sample size of 5000 cases. The poor predictive power of the Kulldorff spatial scan statistic has been noted before: areas with a low incidence rate (far below the global mean) can be included in the cluster area and the local average within this cluster remains sufficiently elevated [12,37]. The BN method is based on the number of k enclosed disease cases which influences greatly the power and therefore the detection rate and the predictive power of the test. At census tract level, the BN method had a mean DR for both risk magnitudes that was lower than that of Kulldorff’s spatial scan statistic for the k-threshold nearest to true number of enclosed cases. Nevertheless, the BN method had a slightly better predictive performance than Kulldorff’s spatial scan statistic because it produced far less FPs. This, however, turns out to be particularly distinct for the urban cluster scenario in females for a RR_2_ = 4.0 at community level, where a power and DR of >90% were achieved with only minimal increased FPs as compared to the Kulldorff spatial scan statistic. But this was only accomplished with the choice of k that was nearest to the true number of observed cases (k = 30).Costa & Assuncao [38] reported in their comparison of the Kulldorff spatial scan statistic and the BN method that the methods perform similarly in urban settings with a sufficiently large background population but show major differences in sparsely populated areas. We observed this pattern in particular in the female urban cluster but not for the male population because the k-threshold was far different from the true number of enclosed cases.

The LMI method level showed the weakest ability to identify clusters and had the lowest ability to predict the cluster correctly (compared to Kulldorff spatial scan statistic and the BN method for best k-threshold). These findings confirm previous simulation studies [8,11-13] which mentioned that LMI had the poorest performance of the local cluster tests. But for the urban cluster in males an interesting trend was observed: With increasing the risk it was the only local cluster test where the increases of TPs were accompanied by a decrease of FP. However, this could be only observed in the urban cluster realization in males, denoting that the applied version of LMI is sensitive to the small number problem. Therefore, it appears reasonable to include a modified version of the LMI in an R package that adjusts for heterogeneous population densities as proposed in [39]. At the community level only spatial outliers are detected, and the exact version of the test was applied because only few spatial neighbours exist which makes the normality assumption arguable. For a twofold elevated cluster risk, the risk realization in the community is too small to be detected as a spatial outlier unlike for a four-fold risk increase: here the LMI had a greater ability to detect the cluster community than the BN method.

### Bayesian smoothing methods

The use of Bayesian smoothing methods for cluster detection are generally characterized by a decision whether the DR should be maximised or the FPR should be minimised for a specific RR cut-off that serves as threshold value for defining a cluster. The results of the Bayesian models are discussed with the objective of evaluating the DR of each of the four models at a minimum FPR (or high specificity).

The global models (PG and log normal) showed poorer test accuracy than the local models and the differences between these two global models were not very distinct. The global models have no definition of a spatial neighbourhood and therefore the risk estimates were smoothed towards the global mean. This is expressed by the course of their ROC curves which were very close to the plot diagonal implying very low test accuracy. This was particularly clear for cluster RR = 2.0, where all models failed to detect the rural cluster in females, possibly due to the low realized risk caused by small sample sizes and dilution effects in this cluster type. For the four-fold RR the test accuracy was augmented for all models, however, the global models were still less accurate than the local ones. Of note, the differences were more distinct than for the two-fold RR realization, implying that the risk signal in the cluster areas were not oversmoothed but rather consolidated. This became particularly apparent in the male urban cluster where the test accuracy of the BYM model exceed that of the local EB model (showing both higher DR together with a lower FPR). This describes also the situation where the BYM model was most powerful, namely moderate sized expected counts (>50) and/or high excess risk [40]. Only few simulation studies are available that compare Bayesian smoothing methods to local cluster tests. The results are consistent with the findings of Aamondt et al. [35] who found in a comparable cluster setting a mean sensitivity between 0-1% for a relative risk of 1.5 but a sensitivity of 85-99% for a RR = 4.0. Similarly, Richardson et al. [40] reported that the BYM model is essentially conservative for moderate relative risks (RR < 2.0) and they concluded that it is nearly impossible to detect localized risk areas if these are not based on a large (RR > 3.0) excess risk or, in the case of a moderate risk (RR > 2.0), on substantial numbers of expected counts of approximately 50 or more. At community level, the expected counts are consolidated by data aggregation although it was noted that the rural cluster in females could neither be detected at RR = 2.0 nor at RR = 4.0, most likely because of the dilution effects in this cluster. The local EB had a mean DR for a RR = 2.0 that was comparable to that of Kulldorff’s spatial scan statistic although with a higher FPR. However, with a four-fold relative risk in a cluster, all Bayesian models had the same higher test accuracy for the male urban cluster, denoting that the expected counts and the relative risk was realized in a sufficient manner This resulted in a performance that was better than that of the local cluster tests. This was also observed for the female urban cluster but the ROC curves were affected by the reduced sample size.

### Strengths and limitations

There are strengths and limitations of this study. A major strength of this study is the modelling of real cancer incidence data in small and large sample sizes with moderately to highly increased risks and at different spatial scales of data aggregation. Furthermore, the modelling of the observed cases as Poisson distributed reflects a more meaningful assumption than a fixed sample size and it increases the applicability of the study results to realistic cancer cluster patterns. This study was limited in terms of the local cluster tests used. Dozens of local cluster tests exist [41-44] and it is possible that other tests may be more successful to detect and predict the clusters. However, a main objective of this study was to apply well-known methods that are available in an open source environment. For creating a continuous risk surface on the basis of area data, Poisson kriging [45] may be used. This technique may result in less smoothing, however, to our knowledge the Poisson kriging approach is not yet properly implemented in an R-package. This applies also to the use of the modified version of local Moran’s I as described in [38] that adjusts for heterogeneous population densities.

## Summary and conclusion

In summary, this simulation study suggests that for the identification of geographic cancer clusters the use of a smaller spatial scale is generally preferable to a higher data aggregation scale. One reason is that cancer is a fairly rare disease and that cancer clusters tend to be limited in time and small in place. Data aggregation results in diluted risks masking the existence of small high risk areas within a larger aggregate of many average risk areas; this impedes the detection of small cancer clusters with a moderate, and even high, risk increase. This is not balanced out by the higher numerical precision obtained by using larger aggregates. With regard to the tests applied, the local cluster tests seem preferable to the smoothing methods for clusters with a moderate risk increase at both spatial scales. Only with very high cluster risks, the local Bayesian smoothing models have lower FPRs for comparable DRs on the aggregate spatial level (community level). It should be noted that, despite the high DR, the Kulldorff spatial scan statistic had a very low predictive ability whereas, by contrast, the BN method showed a good test accuracy but was extremely sensitive to the right choice of the k threshold. Further, the LMI method is expected to probably show a better performance when adjustments for the heterogeneous background populations can be achieved. For the smoothing methods, the study suggests that the local models are generally preferable to the global models.

In conclusion, the commonly used scale of entire communities is too coarse for a systematic cluster monitoring. Smaller scales have to be preferred to enhance more effective cluster detections. We suggest a two-stage approach that combines highly sensitive methods as a first-line screening with methods of higher predictive ability in order to reduce the number of false positive results. For small-scaled data the results of the Kulldorff spatial scan statistic pre-screening could be used to refine the parameter k and then the BN methods appears suitable to re-evaluate the identified clusters. When using a higher data aggregation level, the local EB model appears more suitable. Future research into cancer cluster detection should focus on the numerical and statistical stabilization of the risk measures. Thus, it should be quantitatively evaluated which cancer entities are actually appropriate for a prospective cluster monitoring or whether, in cases of low incidence rates with too low count numbers, cluster monitoring should not be encouraged. In addition, the reduction of risk measure variability needs to be emphasized in sparsely populated areas. Apart from spatial aggregation (only to a degree that avoids too much loss of the risk signal), temporal aggregation, especially in the female population and for rare tumours, should be considered to help stabilize the risk measures.

## Competing interests

The authors declare that they have no competing interests.

## Authors’ contributions

DL and HWH planned and designed the study, VM and OH provided the cancer registry data; DL programmed the R-code, administered the simulations, analysed the data and wrote the first manuscript draft, HWH and EP reviewed the manuscript and provided guidance on all issues related to epidemiological and spatial statistical side of the analysis. All authors contributed to the intellectual content and approved the final manuscript.
